# Plant Beneficial Bacteria and Their Potential Applications in Vertical Farming Systems

**DOI:** 10.3390/plants12020400

**Published:** 2023-01-15

**Authors:** Peerapol Chiaranunt, James F. White

**Affiliations:** Department of Plant Biology, Rutgers University, New Brunswick, NJ 08901, USA

**Keywords:** beneficial bacteria, endophyte, rhizosphere, symbiosis, plant nutrition, plant pathogenesis, vertical farming, controlled environment agriculture, hydroponics

## Abstract

In this literature review, we discuss the various functions of beneficial plant bacteria in improving plant nutrition, the defense against biotic and abiotic stress, and hormonal regulation. We also review the recent research on rhizophagy, a nutrient scavenging mechanism in which bacteria enter and exit root cells on a cyclical basis. These concepts are covered in the contexts of soil agriculture and controlled environment agriculture, and they are also used in vertical farming systems. Vertical farming—its advantages and disadvantages over soil agriculture, and the various climatic factors in controlled environment agriculture—is also discussed in relation to plant–bacterial relationships. The different factors under grower control, such as choice of substrate, oxygenation rates, temperature, light, and CO_2_ supplementation, may influence plant–bacterial interactions in unintended ways. Understanding the specific effects of these environmental factors may inform the best cultural practices and further elucidate the mechanisms by which beneficial bacteria promote plant growth.

## 1. Introduction

According to projections made by the UN Food and Agriculture Organization (FAO), the number of undernourished people is expected to climb steadily from approx. 497 million in 2021 to approx. 682 million by 2050, under a business-as-usual scenario. Although arable land is projected to increase globally, the resulting per capita arable land due to population growth is projected to decrease from approx. 0.215 ha/person in 2021 to 0.176 ha/person in 2050. Paired with this is an increase in agricultural greenhouse gas emissions from approx. 4.77 billion tons CO_2_-eq in 2021 to approx. 5.16 billion tons CO_2_-eq [1]. These projections reflect the business-as-usual practices in agriculture, which rely on the use of synthetic fertilizers and pesticides that are not only energetically intensive to produce, but also lead to downstream pollution via runoff. To meet these challenges, innovations must address both the need to constantly improve crop yield—from the perspective of rising food insecurity—as well as the need to minimize agricultural inputs of energy, nutrients, and water.

Plant growth-promoting bacteria (PGPB) have been explored as a possible solution to reduce the industry’s reliance on agrochemicals while improving crop yield. PGPB are bacteria that reside in or around plants; they can confer growth benefits via various mechanisms, such as supplementary phytohormone production, protection from biotic and abiotic stresses, and improvements to nutrient and water uptake [2,3]. Lab and greenhouse experiments typically show promising growth promotion results when certain strains of PGPB are used as inoculants. However, these results often are not corroborated in field trials, where PGPB inoculations may fail to establish. Inconsistent results may be due to variable biotic and abiotic factors in the field that cannot be accounted for in a lab or greenhouse setting [4,5,6,7].

Vertical farming represents another avenue for technological advancement that can spur improvements in yield, cropping intensity (the number of crop harvests per year), and protection from pests and pathogens, while reducing nutrient and water usage [8,9,10,11]. In this paper, we consider vertical farming to be an indoor agriculture system that uses some level of controlled environment agriculture (CEA) in combination with soilless cultivation [12,13]. The soilless cultivation techniques used in vertical farming do not feature soil as a rooting medium; instead, roots come into direct contact with a nutrient solution either through complete or partial submergence (hydroponics and aquaponics) or periodic misting (aeroponics), or alternative substrates are used [14,15,16]. Because plants do not have to explore a soil medium to scavenge for nutrients, fertigation is more efficient. Nutrients and water are often monitored, controlled, and recirculated in these systems, which further reduces the agricultural input and minimizes runoff risks [17,18].

Together, these two innovations—PGPB and vertical farming—have promising commercial potentials that are currently unrealized due to technological limitations. In the case of PGPB, the limitation in question is the difficulty in establishing PGPB populations in variable field conditions. The major limiting factor of these controlled environment agriculture (CEA) systems is the intensive energy input required for artificial lighting, climate control, and water pumps [8].

Much of the research on PGPB is focused on developing products that can be used in field conditions. However, indoor agriculture environments—due to their control of abiotic and biotic variables—may represent a niche in which PGPB products can thrive and be commercially viable. PGPB products can also supplement indoor agriculture by inducing yield improvements in a non-energy intensive manner. In this review, we will describe the specific benefits and shortcomings of both PGPB and indoor agriculture, how they can benefit each other, and highlight important research that has synthesized the two fields thus far.

## 2. Plant-Associated Bacteria

Bacteria represent about 95% of all microorganisms in the soil, which also includes fungi, protozoa, and algae [3]. Bacterial population dynamics shift drastically depending on the proximity to a host plant; rhizospheric soil typically has a greater concentration of bacteria compared to the rest of the soil due to the presence of plant root exudates in the form of various carbon compounds and organic acids [19,20,21]. These root exudates pose a significant carbon cost to plants and mediate plant–bacterial crosstalk [19,20]. For this carbon expenditure to not constitute a fitness cost, the bacteria must provide growth-promoting functions, which we will discuss in the following sections.

### 2.1. Mechanisms of Bacterial Association with Plants

Plant-associated bacteria may serve three different roles with respect to a host plant: mutualistic, commensal, or parasitic [3,22,23]. Mutualistic bacteria, or PGPB, may be free-living (rhizospheric), endophytic, or they may form unique symbiotic structures, e.g., a nodule formation in legumes by rhizobia [3,22]. Endophytic bacteria are those that reside, for part or all of their life cycle, inside plant tissues [24]. Typically, root endophytes are biphasic, alternating between a rhizospheric phase and an in-planta phase [3,24]. As such, endophytes can be considered a subset of plant-associated soil bacteria.

Mechanisms by which bacteria become endophytic are still unclear, but a recently proposed hypothesis is the rhizophagy cycle. In this model, nutrient-loaded bacteria are attracted by root exudates to become endophytic in plant root tissues, are subsequently degraded by plant-produced reactive oxygen species (ROS) for nutrients, and are then expelled from root hair tips to resume nutrient scavenging or a nitrogen fixation [24,25]. Several nutrients have been found to increase in plants that are engaged in rhizophagy, including macronutrients like nitrogen, phosphorus, and potassium [24,26]. However, it is thought that rhizophagy may be more important in providing immobile and more difficult to obtain micronutrients like zinc, iron, and magnesium [27]. More research is needed to confirm precisely which nutrients are oxidatively extracted from bacteria and which are predominantly obtained by the solubilization of nutrients in soils.

Proper root hair formation is central to the rhizophagy cycle, as root hair tips are the location of bacterial expulsion from the host plant. In certain hydroponic growth conditions, the root hair formation of some plants (i.e., lettuce) is greatly reduced [28,29]. Specifically, adequate P levels in hydroponic solutions may reduce root hair density [30]. It stands to reason that hydroponic growth conditions—which are used in many vertical farming systems—that impact root hair formation may also impair or affect plant–bacterial associations through the rhizophagy cycle. However, this is still an active area of research and the full implications are currently unknown.

Table 1 lists several plant–PGPB systems for which evidence of rhizophagy has been shown in lab conditions. How the plant–bacterial relationships covered here may change with the hydroponic growing conditions is an interesting avenue for research.

### 2.2. Functions of Beneficial Bacteria

**Figure 1:**
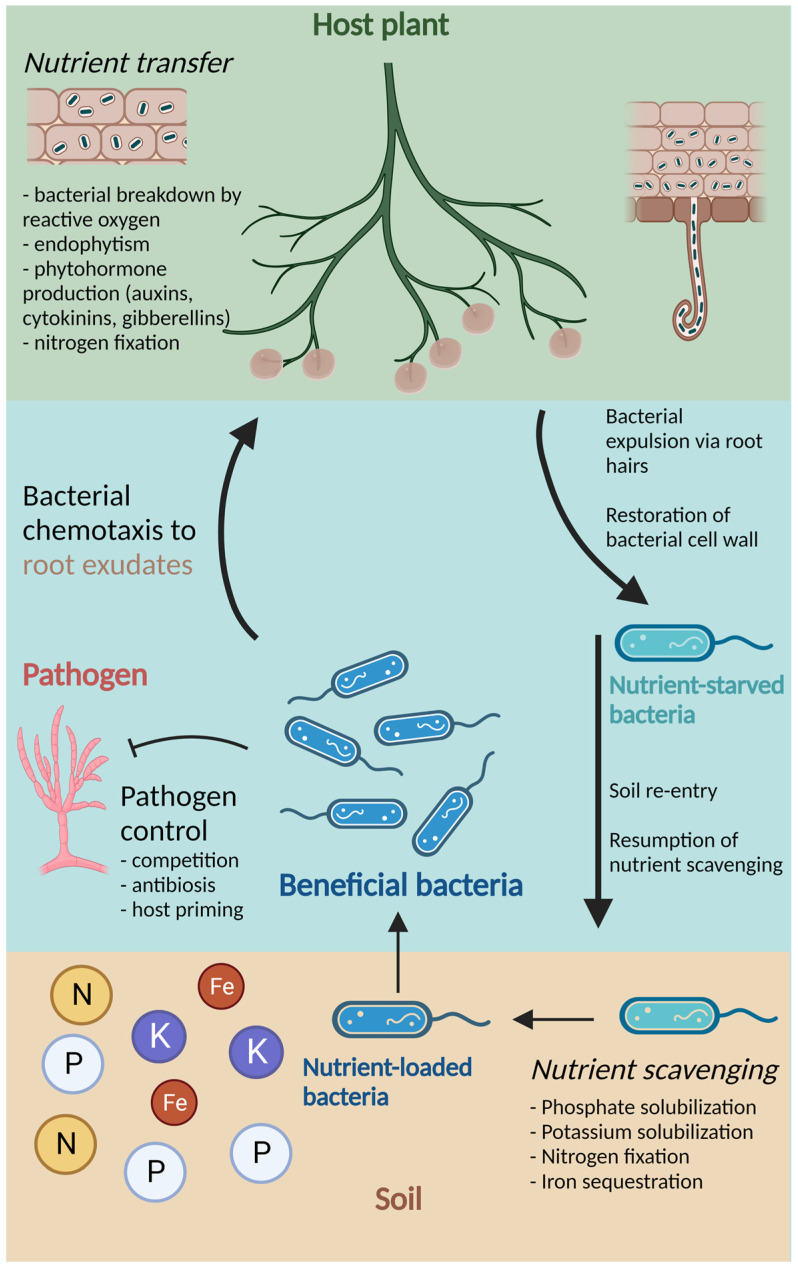
Schematic representation of the growth-promotional and defensive functions provided by beneficial bacteria, including participation in the rhizophagy cycle. The host plant (labeled in green) breaks down soil bacteria with ROS, allowing for endophytism and transfer of nutrients and phytohormones. Following this, nutrient-starved bacteria are expelled via root hairs, where they can restore their cell walls. In soil, bacteria resume nutrient scavenging, which includes phosphate and potassium solubilization, nitrogen fixation, and iron sequestration. Nutrient-loaded bacteria (labeled in blue) are subsequently attracted back to the host plant via root exudates, where they are degraded by ROS and nutrient transfer can occur again. Throughout this cycle, beneficial bacteria may also participate in pathogen control through competition, antibiosis, and priming of the host plant’s resistance. Created with BioRender.com.

Endophytes that participate in the rhizophagy cycle may differ from rhizospheric bacteria in the specific functions that contribute to improved plant nutrition. Other beneficial functions, however, are common between endophytic and rhizospheric bacteria. For example, phytohormone production, abiotic stress relief, and protection from pathogens can be attributed to both endophytic and rhizospheric bacteria, independent of lifestyle [3,22,24]. A schematic representation of the important PGPB functions, including participation in the rhizophagy cycle, is shown in Figure 1.

#### 2.2.1. Biological Nitrogen Fixation

Beneficial bacteria have been shown to provide various benefits to plant nutrition, most notably with macronutrients such as nitrogen, phosphorus, and potassium, but also with certain micronutrients such as iron, which is an essential component of chlorophyll. Nitrogen is often a growth-limiting nutrient for plants; deficiencies can result in a reduced photosynthetic rate, early plant senescence, and the degradation of nitrogen-based enzymes [42].

Biological nitrogen fixation (BNF) refers to the microbial process that converts atmospheric dinitrogen (N_2_) to plant-usable ammonia ions (NH_4_^+^), which can be absorbed by plants [43]. BNF is a highly energetic reaction that requires 16 molecules of ATP for N_2_ breakdown and an additional 12 molecules of ATP for NH_4_^+^ assimilation and transport [44]. The three classifications of diazotrophs (nitrogen-fixers) are free-living, associative, and symbiotic N-fixers [44]. A limited diversity of all free-living and symbiotic microbes possesses the nitrogenase enzyme complex, which is necessary for BNF [45,46,47]. Most diazotrophs possess similar nitrogenase enzymes, which are highly sensitive to inactivation and destruction by oxygen [44,48]. As such, diazotrophs typically have some adaptations that prevent oxygen damage to the nitrogenase complex while simultaneously allowing access to sufficient oxygen to meet the high energy requirements for N fixation.

Positive results have been documented with the associations between lodgepole pine (*Pinus contorta*) and *Pseudomonas spp*. [49]; poplar (*Populus trichocarpa*) and various endophytes [50]; sugarcane (*Saccharum officinarum*) and *Acetobacter diazotrophicus* [51]; wheat (*Triticum aestivum*) and *Klebsiella pneumoniae* [52]; rice (*Oryza sativa*) and *Herbaspirillum seropedicae* [53,54]; and *Setaria viridis* and *Azospirillum brasilense* [55].

The commercialization of N-fixing bacteria is a popular idea, one that is reflected in the large market share that N-fixing biofertilizer products hold within the global biofertilizer market. In 2021, N-fixing biofertilizers hold 79% of the market share of all biofertilizers [44]. Europe and North America dominate the global biofertilizer market, with companies such as BASF (Germany), Bayer (Germany), Isagro (Italy), Valagro (Italy), Koppert Biological Systems (the Netherlands), Acadian Seaplants (Canada), and Kula Bio (the USA) leading the way [56]. *Rhizobium*, *Azospirillum*, and *Azotobacter* are among the common bacterial genera that have been designed as commercial N-fixing biofertilizers [44,56].

Although there have been and continue to be applications for diazotrophs in traditional field agriculture, their usefulness in soilless systems is not as well explored. In these systems, the N supply is highly controlled and often can be supplied at an optimal rate [57]. Several studies have shown that, despite the adequacy of the mineral N that can be supplied in soilless systems, diazotrophs may still contribute by decreasing the amount of chemical input of N fertilizer. In a study with common bean, plants in the treatment groups inoculated with *Rhizobia* spp. and irrigated with a N-free nutrient solution sustained no signs of N-deficiency throughout the growth cycle [58]. Another study with hydroponic bean found that a rhizobial inoculation led to the successful nodulation and sustenance of normal N levels in tissue, but only when the inorganic N supply was restricted [59]. In both of these studies, complications with cation uptake (resulting from the absence of the NO_3_^−^ anion from the nutrient solution) led to smaller plants in general, but the N demand was satisfied [58,59]. For N-fixing bacteria to gain a more widespread commercial use in soilless systems, electrochemical imbalances resulting from reduced-N nutrient solutions should be resolved, and applications to non-leguminous plants—specifically, common hydroponic plants such as lettuce, tomato, and other leafy greens—should be further researched and developed. To this end, there have been several recent studies that document the positive effects from a diazotroph inoculation for such plants. Foliar applications of *Azotobacter* in hydroponic lettuce have led to increased yield and photosynthetic pigments, even under normal N fertigation levels [60]. *Gluconacetobacter diazotrophicus* has been used to increase the growth of both lettuce and tomato in hydroponic conditions, though growth improvements can be ascribed partly to the hormonal regulation and nitrogen use efficiency [61,62].

#### 2.2.2. Other nutrient Benefits

Besides nitrogen, other well-studied nutrition benefits provided by bacteria include improved phosphorus, potassium, and iron uptake. Phosphorus is a commonly limited macronutrient in soils; deficiencies can lead to impairments in several phosphate-involved metabolic pathways, such as membrane synthesis, nucleic acid synthesis, and enzyme activation [63]. Potassium is another macronutrient in soils and is necessary for the activity of a plethora of enzymes involved in photosynthesis, carbon synthesis, and protein synthesis [64,65].

Phosphorus uptake can be improved by bacterial activity. Bacteria can transform insoluble forms of inorganic and organic phosphates into soluble forms that can be absorbed by plants [66,67,68]. The mechanism behind inorganic phosphate solubilization lies in the bacterial production of organic acids. Gluconic acid, the most prominent of these, chelates cations bound to phosphate, effectively liberating the phosphate anion for plants to absorb [69,70]. Organic phosphorus can be mineralized by the action of enzymes such as acid phosphatases and phytases [71,72]. The solubilization of potassium is thought to employ a very similar mechanism using organic acids as well [64].

The vast majority of hydroponic systems use inorganic fertilizer salts in the nutrient solution, in which case the phosphate- or potassium-solubilizing function of PGPBs seems largely irrelevant [30]. However, there is considerable interest in aquaponics as a sustainable agriculture solution that integrates aquaculture (fish production) with hydroponics. In these systems, the organic waste from fish production—consisting of organic forms of nitrogen and phosphorus—is used to feed hydroponic plants [73]. Theoretically, it is possible to employ nutrient-solubilizing PGPB to more efficiently recycle fish waste into inorganic, plant-available nutrients. Several studies have observed improvements to the plant availability of phosphorus, potassium, and micronutrients by using *Bacillus* spp. or other nutrient-solubilizing bacteria [74,75,76]. Thus, nutrient-solubilizing PGPB may play an important role in optimizing nutrient reuse efficiency in aquaponics.

Iron is an essential micronutrient for plants, as it is an enzyme cofactor involved in many metabolic processes; deficiencies in iron can lead to disruptions in respiration and photosynthesis, eventually leading to chlorosis [77,78]. Iron is abundant in most types of soils, existing as Fe_2_^+^ or Fe_3_^+^, with the latter often forming insoluble ferric oxides in high pH soils [78]. In response to Fe deficiency, plants can release protons to acidify soil, liberating Fe from oxides and improving the solubility of Fe [79]. Plants can also produce phytosiderophores, organic substances which can bind and deliver Fe directly to root cells [78]. These two methods of Fe acquisition are not very efficient, however. Certain PGPB can produce siderophores that can supplement phytosiderophores; these bacteria-derived siderophores are highly diffusible in the environment and improve iron solubility and uptake not just for the bacteria, but for proximal plant roots as well [80]. A prevailing notion about bacterial siderophores is that they function not only as Fe carriers, but also serve to mediate interactions between bacteria and their plant hosts [80]. Several hydroponic studies have shown siderophore-producing bacteria to improve Fe nutrition in a variety of crops, such as strawberry, tomato, and wheat [78,81,82].

#### 2.2.3. Phytohormone Production

In addition to the direct activity of enzymes, improved plant growth also results from the bacterial production of various growth-regulating phytohormones. These phytohormones include, but are not limited to: cytokinins (CKs), auxins, ethylene (ET), and gibberellins (GAs) [83,84,85]. The various functions of phytohormones are complex and interrelated; hormonal crosstalk between auxin and cytokinin, for example, is responsible for promoting either root formation or shoot formation, depending on the auxin-to-cytokinin ratio [86]. As such, it is not sufficient for a bacterium to produce high amounts of a certain phytohormone in order to confer growth benefits. Instead, plant-produced phytohormone levels must be supplemented with an appropriate amount of bacteria-produced phytohormones [87].

Auxins and cytokinins are prominent growth-promoting phytohormones that function in regulating cell division, cell differentiation, and senescence [87,88,89,90]. Both of these phytohormones are positive regulators of stomatal opening and have been found in various studies to promote plant growth under drought stress [85,91,92,93]. Auxins, the most studied of which is indole-3-acetic acid (IAA), play an important role in root and shoot cell division and gravitropism. Auxins have been shown to induce the emergence of lateral roots by modulating the expression of aquaporin [94]. Additionally, auxin signaling is involved in the formation and maintenance of shoot apical meristems [95,96]. The overproduction of IAA by bacteria has also been linked to an increased 1-aminocyclopropane-1-carboxylic acid (ACC) production and increased ethylene downstream [93].

Cytokinins play a role in many developmental and physiological processes. Although the biology of cytokinins is complex and many genes from many different gene families influence the synthesis and transport of cytokinins, overall cytokinin growth promotion function includes delaying senescence, regulating apical dominance, and improving grain yield in cereal crops [97,98,99]. Cytokinins are also involved in cell growth and division [100,101]. *Arabidopsis thaliana* mutants deficient in the cytokinin receptor activity were found to have impaired root growth, suggesting the important role of cytokinin in root development [102].

Ethylene is a growth and stress hormone in plants that has also been shown to be produced by microbes via the activity of microbial ethylene synthase (MES) [103]. Chang et al. [26] showed that the elongation of root hairs was stimulated by bacteria that produced ethylene in the tips of root hairs. Experiments that were conducted on seedlings where the MES activity was blocked by using a non-functional analogue of arginine (substrate of MES) resulted in the complete failure of root hairs to elongate. In those experiments, blocking plant-produced ethylene had little effect on root hair elongation. Thus, it was posited that root hair growth is largely dependent on the microbially produced ethylene within root hairs where bacteria accumulate.

Gibberellins play an important role in various physiological processes. Among these, gibberellins are involved in altering gene expression to affect seed germination and dormancy, root and shoot growth, and the production of hydrolytic enzymes to regulate the starch content in plants [104,105,106]. Several bacterial genera have been shown to produce gibberellins, including but not limited to: *Bacillus*, *Pseudomonas*, *Acinetobacter*, and *Burkholderia* [107,108,109,110]. Inoculation experiments with several such bacteria have shown promising growth promotion results. For example, radish plants inoculated with IAA- and GA-producing strains of *P. fluorescens* and *B. subtilis* showed increases in root and shoot biomass, photosynthetic pigments, and nutrient content under salt stress [110]. In a different study, GA-producing *B. methylotropicus* was shown to improve seed germination in lettuce, cucumber, soybean, and mustard [111].

Although the value of hormonal shoot growth promotion is obvious for crops grown in soilless culture, it is not as clear whether improved root growth is needed. As a general rule, the root system is not a limiting factor for plants to meet their nutrient requirements in certain soilless systems where nutrients are constantly replenished [30]. However, the use of phytohormone-regulating PGPBs may be useful for crops with valuable root products, such as potatoes, yam, ginger, valerian, etc. [112]. Some studies applied GA and auxin—combined with the use of an aeroponic system—to improve potato yield [113,114]. Integrations of different soilless system designs and PGPB functions may be useful in expanding the range of feasible crops for soilless agriculture.

#### 2.2.4. Abiotic Stress Relief

There are abiotic stresses that are pertinent to vertical farming systems, such as root hypoxia and a high salinity due to the buildup of ions in recirculating water [30]. Root hypoxia may present a risk when hydroponic systems are improperly aerated, which can lead to impaired root respiration and elevated ethylene levels [30,115]. Ethylene, as a gaseous phytohormone, can be transported through the xylem to affect distal plant organs (e.g., leaves, fruit), where it can induce ethylene response factors, which can inhibit cell division and growth [116]. On the other hand, high salinity in hydroponic systems can affect the uptake and translocation of certain anions (such as Ca_2_^+^, K^+^, and NO_3_^−^) due to ionic imbalances, leading to deficiencies that can affect growth and functioning [30,117]. In general, abiotic stresses also result in the accumulation of reactive oxygen species [118].

Abiotic stress can induce stress response signaling in plants that involves a variety of signaling molecules. A prominent mechanism by which PGPB reduce abiotic stress responses involves the enzyme 1-aminocyclopropane-1-carboxylate (ACC) deaminase. In response to any of the aforementioned abiotic stressors, the ethylene signaling pathway is engaged. In this process, the enzyme ACC synthase is upregulated. ACC synthase converts S-adenosyl-methionine, a conjugated form of methionine, into ACC. ACC is converted to ethylene by ACC oxidase. As mentioned, part of the plant response to elevated levels of ethylene includes the inhibition of growth [115].

Certain PGPB natively produce ACC deaminase. Before ACC is converted into ethylene, a portion of it can be transferred between the host plant and its endophytic partner via root exudation. ACC deaminase activity allows bacteria to metabolize ACC. The enzyme allows ACC to be cleaved into ammonia and α-ketobutyrate [103]. This process effectively reduces the amount of ACC that is converted into stress ethylene and growth-inhibitory ethylene response factors [103,116].

PGPB that produce IAA may confer greater plant benefits if they also produce ACC deaminase. In the model of IAA and ethylene crosstalk proposed by Glick (2014), IAA induces the post-translational upregulation of ACC synthase, which could lead to elevated levels of ethylene [103]. However, the production of ACC deaminase can negate this increase in the ethylene levels, essentially allowing the IAA produced by the PGPB to continue promoting plant growth via an increased shoot cell division, gravitropism, and lateral root formation [94,95,96,103].

Other ways that PGPB can combat the aforementioned stressors include the synthesis of extracellular polymeric substances (EPS), the synthesis of osmolytes, and the upregulation of antioxidant enzymes [118]. EPS are negatively charged polymers that can bind excess Na^+^ that may have accumulated in recirculating systems, facilitating Na^+^/K^+^ osmotic balance [118,119]. Osmolytes are plant metabolites whose production can be bacterially induced; these metabolites alleviate salinity stress as well by improving the cellular retention of water [118]. PGPB can also upregulate a variety of antioxidant enzymes, which can help alleviate both hypoxic stress and salinity stress by detoxifying reactive oxygen species [118].

#### 2.2.5. Pathogen Control

PGPB may function as biocontrol agents in response to pathogens. This is achieved through competition for the niche within a host plant or substrate, the secretion of antibiotic compounds and lytic enzymes, and the induction of the host’s systemic resistance [120,121,122].

Broadly speaking, PGPBs produce a wide range of metabolites that can provide pathogen-antagonistic functions: these natural products may be synthesized by multi-domain enzyme complexes and include nonribosomal peptides (NRPs), polyketides (PKs), and ribosomally synthesized and post-translationally modified peptides (RiPPs) [123,124,125]. NRPs are a structurally diverse class of secondary metabolites that are produced by multimodular NRP synthetases [125,126]. RiPPs are produced as linear peptides that are subject to a large variety of post-translational modifications, resulting in a great diversity of the structures [124]. PKs, produced by PK synthases, are another class of natural molecules that may have anti-microbial properties [127]. Due to the great diversity of structures that can result from the biosynthesis of NRPs, PKs, and RiPPs, these compounds may be promising in the pursuit of novel antibiotic compounds for potential use in vertical farming systems, especially to counteract antibiotic-resistant bacteria [123,124,125]. Recent progress made in this field has seen the use of microbial co-cultures to produce novel PKs [127] and genetics-based approaches to identify novel antifungal NRPs, PKs, and RiPPs [128,129].

Antibiotic compounds produced by PGPB can be categorized into two groups: volatile antibiotics and diffusible antibiotics [122]. Hydrogen cyanide and dimethyl disulfide are examples of volatile antibiotics [122]. Hydrogen cyanide has been observed to inhibit various pathogens including the ascomycete *Thielaviopsis basicola* in tobacco plants [130], *Agrobacterium tumefaciens* and the nematode *Meloidogyne javanica* in tomato [131], and even the insect *Galleria mellonella* [132]. Diffusible antibiotics, on the other hand, are solid or liquid compounds at an atmospheric temperature and pressure, and may include 2,4-diacetylphloroglucinol (DAPG), phenazines (PHZ), alkanes, and hexanoic acid [122,133]. PHZs, as an example, have been shown to compromise the cell membranes of plant pathogens [134], and hexanoic acid has been shown to inhibit *Botrytis cinerea* in tomato [135].

Several lytic enzymes produced by PGPB include cellulases, proteases, and chitinases [121]. These enzymes can affect the cell wall integrity of pathogens [136]. Strains W81 and 34S1 of *Stenotrophomonas maltophilia* have been shown to have biocontrol activity against *Pythium ultimum* and against summer patch disease, respectively, due to the action of extracellular enzymes such as chitinases and proteases [137,138].

Pathogens may be excluded from the plant host by PGPB competition for nutrients or for colonization in host roots [139]. Nutrient competition by PGPB may involve iron sequestration via siderophores; this effectively reduces the available iron for plant pathogens [122]. Although not always necessary, the colonization ability can correlate with the biocontrol ability of PGPB [122]. A mutant study found that *Pseudomonas chlororaphis* deficient in root colonization became less effective at controlling *Fusarium oxysporum* in tomato, despite producing normal levels of PHZ [140].

Lastly, PGPB can trigger the accumulation of defensive compounds in their plant hosts. Termed ‘induced systemic resistance’ (ISR), this process involves complex hormonal and molecular control, with jasmonic acid and ethylene as key players [141,142,143]. There are a number of reviews that describe ISR in greater detail, so here we only present a brief overview [143,144,145]. As PGPB colonize the plant roots, ISR is initiated by elicitors, such as microbe-associated molecular patterns, lipopolysaccharides, antibiotics, DAPG, and flagella, to name a few [143]. Elicitors are perceived by pattern recognition receptors (PRRs) and work redundantly to trigger defense mechanisms throughout the whole plant. Typically, the defense is improved via the increased expression of jasmonic acid- and ethylene-dependent defense genes and the increased deposition of callose at plasmodesmata; the latter effect helps to prevent the movement of pathogens between cell junctions [143]. The hormones involved in ISR include jasmonic acid, ethylene, auxin, and nitric oxide [144]. Associations between PGPB and their hosts may involve the hijacking or suppression of host defenses, which allows PGPB to establish in plant roots [143,144].

### 2.3. Field Inconsistencies of PGPB

Despite the benefits conferred by PGPB in laboratory and greenhouse environments, the results in the field remain variable and inconsistent. There are several factors that influence the efficacy of PGPB in the field. These include variable soil abiotic and biotic environments, incompatibility between a host plant’s genotype and a PGPB strain, unforeseen interactions between a PGPB and the existing soil microbial community, and difficulties in the storage and transportation of PGPB products [146,147,148].

Multiple solutions have been proposed to address these challenges. *Bacillus* spp. are commonly used as biofertilizer products because of their ability to form endospores, whose stability and inertness make them suitable for long-term storage and transport [149]. However, there are many other genera of PGPB that promote growth but do not form endospores. Furthermore, *Bacillus* spp. are not compatible with all crops. Delivery methods involving seed coatings are currently used to some success for non-*Bacillus* PGPB; however, artificial seed coats may reduce the microbial viability, and coated seeds tend to have a shorter shelf life [150,151,152].

Soil and plant inoculation are alternative methods of PGPB delivery. Soil inoculation involves adding liquid or granular inoculants into the substrate, which may allow for a sufficient colonization by the PGPB [153]. However, adding PGPB to a soil environment can lead to unforeseen antagonisms between the PGPB and the soil microbiome. Plant inoculation involves root dipping or foliar spray. Both soil and plant inoculation require high amounts of inoculant and may not be feasible for large-scale agriculture [153]. Additionally, these inoculation methods can have varying degrees of success due to weather conditions. Precipitation, temperature, and humidity may affect the viability of inoculants as well as their ability to effectively colonize plants. The duration of exposure between the plant and an inoculant applied via foliar spray can be adversely affected by rain, for example.

Since plants–PGPB have proven to be highly dependent on environmental factors, and traditional field agriculture has many uncontrollable variables, it stands to reason that PGPB technology might be better suited to vertical farming systems, where there is much greater control over certain variables in the growing environment.

## 3. Vertical Farming

Vertical farming gained mainstream interest following the publication of the book by Despommier [9], who theorized the upscaling of arable land by building upwards. By constructing tall, climate-controlled buildings with many levels of growing space stacked vertically, the challenges to traditional farming can be negated [9]. Whether or not Despommier’s ideas for so-called vertical farming are economically feasible has been a subject of much debate [154]. Here, we present an overview of vertical farming setups, comparisons between the different vertical farming types and soil and hydroponic agriculture, and a summary of the advantages and challenges to vertical farming.

### 3.1. Vertical Farming Systems and Setups

Vertical farming systems fall into one of two main types. The first type comprises systems where plants are grown on horizontal growing spaces that are stacked skyward. The second type involves growing plants on vertical surfaces. Stacked horizontal systems can further be differentiated by the type of hydroponic technology used, the implementation of growing level rotation (to ensure adequate sunlight for lower growing levels), and whether or not growing levels are isolated from each other. Vertical growth surfaces can be grouped into two subcategories: green walls, where plants are grown on the side of buildings, and cylindrical growth units, where plants are grown around upright cylindrical units containing a central nutrient supply. All vertical farming systems can vary in the extent to which the growing environment is controlled; in other words, whether or not controlled environment agriculture (CEA) is used. Vertical farms without CEA can be implemented in glasshouses, where access to sunlight is an important consideration. For a more detailed breakdown of vertical farming types, see Beacham et al. [154].

Different vertical farming systems may differ in the amount of monetary investment required, the energy required to operate, the potential crop productivity, whether or not the placement of the vertical farm is important, and the types of crops that are suitable to grow in the systems. Table 2 provides a summary of these factors for different vertical farming systems, as well as a comparison between soil-based agriculture and traditional (non-vertical) hydroponic systems.

### 3.2. Advantages of Vertical Farming

The nominal advantage of vertical farming is its ability to exploit a vertical space. It is estimated that the productivity of each acre of indoors vertical farming is equivalent to 4–6 acres of traditional farming, depending on the crop [156]. Part of this productivity increase is due to year-round harvesting, but improved space efficiency is also an important factor. A theoretical 37-story, 0.93 ha vertical farm as conceptualized in Banerjee and Adenaeuer [157] is capable of supplying 15,000 people with 2000 kcal of nutrition per day, in the form of potatoes, spinach, lettuce, cabbage, peas, tomatoes, etc. In this theoretical example of a vertical farm, the yield per hectare is doubled compared to traditional agriculture due to technological improvements (e.g., closed environment and LED lighting); however, additionally factoring in production stacking and the yield per hectare can be improved by an estimated factor of 516 [157]. A more recent review by O’Sullivan et al. collated publicly available data to estimate, for lettuce and leafy greens, an average annual yield of 2 kg/m^2^ for field systems and 100–200 kg/m^2^ for vertical systems [161].

Some vertical farming systems feature controlled environment agriculture (CEA). CEA allows for growers to completely control and monitor important variables, such as light (the intensity, wavelength, and photoperiod), air (the wind velocity and ambient air temperature), and water (the pH level, electrical conductivity, nutrition, dissolved oxygen levels, and water temperature) [158]. The possibility for virtually complete control of a plant’s abiotic environment provides a range of economic, environmental, and growth advantages. By isolating a crop from changes in climate and nutrition, CEA can streamline growth and improve crop productivity, while allowing for year-round cultivation [159,162]. In addition, CEA allows for the production of crops in areas with extreme climates or even in outer space missions. A pioneering example of CEA is the EDEN ISS project near the German Neumayer III station in Antarctica. EDEN ISS is a CEA facility with external air temperatures as low as −43.5 °C [163]. Despite this, it was able to produce 268 kg of fresh edible biomass of tomatoes and cucumbers (105.4 kg), lettuce (56.4 kg), leafy greens (49.1 kg), tubers (26.8 kg), herbs (12.2 kg), and miscellaneous crops (18.4 kg) [163]. State-of-the-art CEA facilities such as the EDEN ISS feature sensors and automated control systems for the fine regulation of pH, nutrition, and LED lighting [158,163].

Some vertical farming setups also recirculate nutrient solutions in a hydroponic system. There are multiple types of hydroponic systems; commercially, the nutrient film technique (NFT) and deep-water culture (DWC) are most commonly used, with aeroponic and aquaculture techniques also growing in popularity [164,165]. NFT recirculates a thin film of nutrient solution for a constant flow in the root zone, while DWC submerges roots in a deep reservoir [57]. The recirculation of a nutrient solution offers multiple benefits, including the precise control of nutrition, reduced water consumption, and reduced nutrient usage, which reduces fertilizer runoff and downstream eutrophication [166,167]. Hydroponic recirculating systems are estimated to accrue irrigation water savings by 80–90% and fertilizer savings by 55–85% [165,168]. Despite the decrease in input, crop productivity can be maintained or increased in CEA hydroponic systems. The life cycle of hydroponic lettuce, for example, is much shorter and a full crop can be harvested every 35–40 days in an NFT system [165].

The cultivation of plants in an indoor environment greatly reduces the presence of soil pests and pathogens. In addition, various treatments can further reduce the possibility of a pathogen outbreak in a hydroponic system. The use of filters with small pore sizes can prevent the introduction of some pathogens into a closed hydroponic system; however, filters with larger pore sizes are also used to remove any precipitates in the nutrient solution, which can enhance downstream disinfection methods [57,169]. Heat and UV treatment of a nutrient solution can be applied to further ensure sterility, although a heat treatment would require a subsequent cooling treatment before the exposure of plants to the nutrient solution [57,164]. UV systems also have a disadvantage of precipitating Fe-EDTA in nutrient solutions, which would deprive plants of necessary iron if Fe-EDTA is not supplemented downstream [57]. Chemical controls using different fungicides are also a possibility, but the risks involved include fungicide resistance and the unwanted elimination of endophytic fungi [164,170]. Other chemical disinfection methods involve the use of oxidizing agents such as hydrogen peroxide and sodium hypochlorite [160].

By virtue of having a much smaller land footprint and being possible to adopt virtually anywhere regardless of climate, vertical farming facilities can be constructed in heavily populated urban centers. Having food production facilities close to their consumer base can drastically reduce the costs and CO_2_ emissions associated with transportation and food storage [159]. Furthermore, food spoilage resulting from long-distance transport is also expected to decrease. Public health can benefit from a fresh and year-round supply of fruits and vegetables. If done correctly, vertical farming can produce vegetables more consistently and with the minimal use of pesticides, thus improving public health.

Resiliency in the face of climate change is an important advantage of vertical farming. A variety of negative effects can result from climate change, including unpredictable local weather events, expanding pest and pathogen ranges, increased occurrences of droughts and floods, and heightened heat stress [171,172]. A traditional farm can attempt to combat these stressors through the increased application of pesticides and increased irrigation and fertilization, but these approaches can be wasteful and ultimately environmentally destructive. Genetic approaches to breeding hardier plants for a more inhospitable future is a possible solution, but these approaches are hindered by the complex genetics underlying (and often linking) plant stress tolerance and yield [173]. A vertical farm, by isolating a crop from its abiotic and biotic environment, entirely circumvents these emerging issues.

### 3.3. Challenges to Vertical Farming

Despite the myriad benefits that vertical farming offers for the environment, grower, and consumer, there remains challenges that currently prevent its mainstream adoption. Among these are technological challenges, initial financial costs for setup, and the simple fact that some of the biological needs of a crop—namely light, CO_2_, and space, which are supplied either freely or at low cost in traditional agriculture—must now be supplied at a higher cost [157]. Additionally, there are other operational factors that can be difficult to implement, such as maintaining a standardized nutrient solution, optimizing the light spectra per crop, and pathogen avoidance in a closed hydroponic system.

Importantly, there are a number of crops that are not suitable or economically feasible for vertical farming systems. Examples include staple crops such as corn, soybean, wheat, rice, and potato [174]. These crops, while extremely important for diets globally, are energy-intensive and have a low ratio of salable to non-salable plant parts, making them an economically unattractive choice for vertical farms [13]. In vertical farms that use CEA, all the inputs to plant growth come at a cost, so the most profitable plants are leafy greens, herbs, and some fruits such as tomatoes, peppers, and strawberries [174]. As such, the commercial application of vertical farming technology is mostly limited to these crops. Certain technological advancements that improve plant output with a minimal cost may expand the range of economically feasible crops. We have discussed one such example: the use of phytohormone-regulating PGPB in aeroponic potato production to improve yields [113,114].

Indoor growing reduces the amount of natural light available for plants; this reduction is further compounded in vertical farming facilities located in urban areas, where tall buildings cast shade [175]. Thus, vertical farming requires a large amount of supplementary light-emitting diodes (LED). The energy requirement for LEDs can be enormous; Perez [175] estimated that if the entire of the United States agriculture industry were to convert to vertical farming, the energy required for lighting alone would be eight times the annual energy production of power plants in the country. Despite this, LED is considered an important technological advancement in horticultural lighting, boasting advantages such as the ability to finely control light levels, intensity, and spectral output. Compared to earlier lighting technologies, LEDs are longer lasting and can be deployed in closer proximity to plants (due to lack of heat radiation). Advancements in LED lighting have focused on optimizing energy efficiency and cost savings; these improvements are particularly important for reducing the costs for vertical farming [176].

Perhaps the most important challenge lies in the high setup and operational costs of vertical farms. In a model comparing the costs of a theoretical semi-closed greenhouse and an equivalent vertical farming system in Quebec (each with a 1171 m^2^ growing space, suitable to supply around 6250 kg of lettuce per year), the capital expenses required to construct the vertical farming facility was estimated at USD 587,527 [177]. The capital expense of indoor LED lighting was USD 203,095, or 34.6% of the total cost; grow unit racks and hydroponic systems represented the next largest expenses at USD 98,375 (20.5%) and 85,492 (17.8%) respectively. Besides the capital expenses, operational costs each year were estimated to be USD 208,382; the main contributors to this figure include labor, at USD 89,774 (38.8%), and electrical costs—for lighting, HVAC, and miscellaneous—at USD 53,548 (25.7%). These costs can be offset by the aforementioned advancements in LED efficiency, and technological and policy changes can impact the prominence of solar- or wind-derived energy, which could improve the appeal of vertical farming.

Besides these concrete costs to implementing vertical farming, there are less predictable factors to consider as well. In closed-loop, recirculating hydroponics, nutrient solutions must be constantly monitored and adjusted. As the nutrient solution is circulated, its composition may change over time due to chemical reactions (complexation and precipitation) and a crop’s differential uptake of different nutrients. For example, Na^+^ and Ca^2+^ are absorbed more slowly by most plants, leading to nutrient imbalances [57,166]. In a recirculating hydroponic system, these nutrient imbalances build up over time to elevated concentrations that can become phytotoxic. Monitoring may be based on individual nutrient concentrations using in-line sensors, but this method is relatively expensive. More common is the practice of measuring the electrical conductivity (EC), which can gauge overall salinity in a nutrient solution, but does not indicate individual nutrient compositions [178]. Adjustments based on either electrical conductivity or individual nutrient compositions are therefore essential to prevent a salt buildup.

Another potential issue is the outbreak of pathogens. Environmental conditions in a vertical farm or greenhouse, such as high ambient temperatures, a high relative humidity, and close plant spacing, make them particularly amenable to the spread of disease [179]. Although we have mentioned various measures that can be taken to prevent pathogen entry into a recirculating hydroponic system, it is realistically impossible to exclude pests and pathogens from entering greenhouses and controlled environment vertical farms [180]. The closed loop of hydroponic systems, along with the nutrient-rich composition of the feed, are factors that make such systems particularly vulnerable to the buildup and spread of pathogenic agents. Of particular concern is the biofouling and eventual clogging of pipes by biofilm-forming bacteria [169]. Antibiotics can be added to the nutrient solution to combat these issues, but studies have observed their uptake and phytoaccumulation by hydroponically-grown plants, as well as the creation of antibiotic-resistant bacteria [181,182,183,184]. Additionally, an antibiotic treatment can affect not only pathogenic microbes, but also any mutualistic or commensal bacteria [185]. Novel approaches to addressing biofouling—and also certain plant pathogens—may involve the use of quorum quenching bacteria or enzymes; this technique can be used to disrupt quorum sensing behavior, which is the basis for the formation of biofilms [186,187]. Table 3 is a summary of the aforementioned advantages and challenges to indoor vertical farming, assuming a typical closed-loop hydroponic system is used.

## 4. Intersection of PGPB and CEA

PGPB in indoor growing systems have seen some, but not widespread commercial use. There are currently some products on the US market that are advertised as compatible with hydroponic systems, but such products are typically developed with intent to use for field-based growing. A major limitation to PGPB establishment in field trials is the variability of the outdoor soil environment. In this regard, the nominal advantage of CEA is the ability for growers to manipulate the growing environment [158,159]. Given that many plant–PGPB relationships require specific environmental conditions to flourish, it stands to reason that CEA facilities are better suited to the successful implementation of PGPB programs, compared to open-field, traditional agriculture systems. Despite this, research in this area is still lacking. We provide below a short discussion of plant–PGPB relationships in soilless systems, as well as an overview of several factors that can influence plant–PGPB relationships in such systems.

### 4.1. Microflora in CEA Systems

Certain genera of bacteria have been documented to promote the hydroponic growth of a variety of crops in a hydroponic culture. Among these are *Pseudomonas*, *Bacillus*, *Enterobacter*, and *Streptomyces*, which confer biocontrol benefits to a variety of plant pathogens [164]. *Pseudomonas* has been documented to provide biocontrol of several *Pythium* species for cucumber and tomato [191,192,193]. Certain *Pseudomonas* strains isolated from a hydroponic system for tomato have been shown to produce auxin [194]. *Bacillus* has been shown to control *Pythium* spp., *Cryptococcus coccoides*, *Fusarium oxysporum* f. spp., and *Rhizoctonia solani* for tomato, chrysanthemum, peppers, and lettuce [194,195,196,197,198]. Inoculations of tomato with *Bacillus amyloliquefaciens* can increase yield; however, the benefits were dependent on an open-loop hydroponic system, and inoculations seemed to be harmful in a closed-loop system, which may suggest that potential nutritional imbalances in a system may affect the outcome of plant–PGPB relationships [199].

Beyond the discussion of single-strain isolations, however, is the approach of designing synthetic microbial communities (SynCom) [200,201]. It is widely acknowledged that moving agriculture indoors will result in a significant loss of endogenous soil microbes, some of which play a major role in suppressing diseases and facilitating plant growth [3,22,23,185]. As a consequence, outbreaks may become more prevalent, and some minor diseases become more harmful in indoor hydroponic systems [185]. Further compounding this problem is the treatment and disinfection that is carried out in some hydroponic systems [57,164,169,185]. The SynCom approach focuses on identifying key microbial members and designing synthetic microbial communities to replicate the functional diversity of the soil rhizosphere [200]. As plant–microbial relationships are further elucidated for specific crops in their natural growing environments, SynCom designs can improve and these may be important in creating soilless, CEA systems that have the same functional redundancy and disease resistance as soil systems.

Attempts to incorporate SynComs into soilless systems should be reconciled with any existing microflora, however. Despite their apparent sterility, even in CEA, microbes quickly colonize certain niches in a soilless system: the substrate, the nutrient solution, and the rhizosphere [185]. These microbes can range from pathogenic to beneficial, and communities are influenced by the type of substrate, nutrient solution, and crop [185]. For example, a rockwool substrate can increase the preponderance of *Pseudomonas* spp. in a tomato-growing system, while peat substrates favor fungi [176]. In any case, managing the microflora of soilless systems should involve the manipulation of substrates to promote the development of disease-suppressive microflora or the combination of disinfection and SynCom design [185,200].

### 4.2. Factors That Can Influence PGPB Success in CEA

Several factors in the CEA system of a vertical farm can influence the plant–PGPB relationship. These factors include the substrate used, root-zone oxygenation, temperature, light quality, and CO_2_ supplementation. Priority effects may also play a significant role in determining a successful PGPB establishment.

#### 4.2.1. Substrate

Contemporary hydroponic systems may use many different types of substrates, such as peat, coconut fiber, bark, wood fiber, and rockwool, to name a few [13,169]. Substrates are required for hydroponic systems, as they serve to anchor roots and provide water and nutrients during the early development of the crop [169]. Different substrates may be composed of a mixture of materials with different physicochemical properties—such as the humidity, potassium content, pH, and electrical conductivity—which can constitute niches for different bacterial members to thrive [202]. These differences are also reflected in the composition of the microbial community of each substrate. Organic substrates have been found through high-throughput sequencing to have more diverse and more stable bacterial communities, compared to mineral substrates [202].

The specific components in a substrate include the composition of peat, composted material, organic material, and inorganic material. A recent study measured the CO_2_ production of 16 different substrate mixtures as a proxy for the microbial activity, to assess the effects of specific substrate components on microbial respiration [203]. White peat is more stimulatory of microbial activity than black peat, for example; this property is attributed to the less decomposed state of white peat, which makes it more amenable to microbial growth [203]. The type of composted material in a substrate mixture may also influence microbial activity, with the same study reporting a greater CO_2_ production attributed to composted bark, compared to green waste compost [203]. In the study, the specific organic materials (coir pith versus wood fiber) and inorganic materials used (perlite) did not result in significant changes to the measured CO_2_ production [203]. Different constituents in a substrate mixture can also affect the physical properties—such as the dry matter content, organic matter content, water capacity, and bulk density—or chemical properties, such as the pH, EC, and the content of macro- and micronutrients [203].

#### 4.2.2. Oxygenation and Flow Rate

Vertical farming systems have an array of soilless growing strategies that can be used, with each strategy providing different levels of root oxygenation. For example, aeroponic culture suspends plant roots in the air, while DWC systems submerge plant roots in the nutrient solution. It is well understood today that insufficient root aeration can be a cause of poor plant productivity, so DWC systems are typically equipped with a means of dispersing oxygen to the root zone through air pumps [204].

Oxygenation can have various physiological and morphological effects on plant roots. Deficiency in oxygen is typically associated with reductions in the total root length and alterations in the root architecture to favor adventitious roots [205]. Besides the effects in the root, the shoot of a plant may also experience changes due to low root-zone oxygenation, including a stomatal closure, the slowing of leaf expansion, and wilting due to an ethylene accumulation [205]. In some plants, aerenchyma–gas-filled tissues which feed oxygen from the oxic shoot to the anoxic root, may also form [205,206]. Plants may also produce toxic compounds such as ethanol, lactic acid, and alanine in response to a low root zone oxygenation [206].

The plant microbiome, likewise, changes in accordance with varying oxygen levels. Oxygen diffused through aerenchyma can end up in the rhizosphere, where an oxygenated zone can develop to favor aerobic bacteria [206,207]. Not all plants, however, are capable of sustaining an aerobic rhizosphere in response to root flooding; a study with wheat found that the rhizosphere oxygen concentration remained low following flooding, with no apparent restoration due to aerenchyma [206,208]. The accumulation of ethanol in roots in response to flooding stress can also theoretically play a role in shaping the microbiome structure [206].

#### 4.2.3. Temperature

Temperature is an important driver of microbiome assembly. The microbiome of wild strawberry populations in North America and Europe, sampled using transects, were found to be highly influenced by temperature [209]. As another example, short periods of heating up to 50 °C can have a significant effect on suppressive soils, causing them to lose their disease suppression due to a reassembly of the soil microbiome to favor heat-tolerant species [210]. In a CEA system, temperature can be more finely controlled, so it is important to determine the sensitivity of the microbiome to daily fluctuations in temperature. In a hydroponic study on rose plants where the daily temperature ranged from 12 to 22 °C, it was determined that these fluctuations did not appreciably affect the bacterial community’s structure [211].

Root-zone temperatures may influence and be influenced by several factors. The volume in which roots are grown can influence the amount of temperature fluctuation that can be expected [212]. Compared to soil, plants growing in smaller containers may experience greater temperature fluctuations [212]. It is possible that variability in temperature fluctuations, dependent on the container’s size, can impact microbiome stability, but research for this particular question is scarce. The temperature of a nutrient solution can also impact oxygen levels, as higher temperature nutrient solutions can decrease the quantity of dissolved oxygen [205]. Different plants may have microbiomes that vary in their sensitivity to temperature fluctuations as well, so additional research in this area is needed.

#### 4.2.4. Light Quality

Although literature is scarce, there is some evidence to suggest that light can impact the plant microbiome. Several studies have observed that UV-B irradiation can impact the microbial community’s structure on leaves [213,214,215]. Light can also impact the temperature of the canopy microclimate, which can influence the phyllosphere microbiome [216]. A study on the effects of high-pressure sodium (HPS) lights and light-emitting diodes (LEDs) on sunflower found that the phyllosphere community may also be altered as a result of the light spectral quality [217]. Different wavelengths of light can affect the production of secondary metabolites, which may play a role in microbial defense [218]. Since a major benefit of LED over HPS lighting is the ability to manipulate light spectra, research on the effects of the spectral quality on plant–bacterial interactions can have important implications for optimizing lighting in vertical farms [176,218].

#### 4.2.5. Root Exudates and Implications for CO_2_ Supplementation

Plant exudate activity is an important determining factor of the microbial community’s composition in soilless growing systems [202,219]. A mutant study of *Arabidopsis thaliana* determined that the biosynthesis of root-exuded coumarin compounds is responsible for the redox-driven maintenance of *Pseudomonas* populations in hydroponics [201]. The roots of cucumber plants grown in rockwool are usually colonized by *Pseudomonas* spp., which provides antagonism to *Pythium aphanidermatum* by limiting the amount of exudates present on the roots [219]. A study on aeroponic lettuce determined that the root microbiome is distinct from the microbial community in the recirculating nutrient solution, suggesting that root exudates impose a strong selective pressure on the bacterial members that colonize the plant [220].

Root exudates definitely play a strong role in selecting the plant host’s bacterial partners, but the degree to which plants actively control exudation to select for specific microbes is still an open question [221]. An important environmental factor that can affect the exudate activity is the practice of CO_2_ supplementation in vertical farming systems, which can be performed in aeroponic systems in order to counteract high root zone temperatures [222,223]. Such practices can impact root exudation, with many studies reporting that elevated CO_2_ results in increased root exudation [224,225,226,227]. A study of the wheat microbiome showed that elevated CO_2_ increased the relative abundance of bacteria and influenced the abundance of genes encoding enzymes, transporters, and secretion systems [227].

#### 4.2.6. Plant Age

Finally, plant age can have a strong influence on the stability of its microbiome and, therefore, the effectiveness of a bacterial inoculum. Tomatoes grown in recirculating nutrient solutions have been observed to establish a robust microbiome merely hours after planting, with the microbiome being resistant for over 12 weeks [228,229]. Inoculations are more effective when performed early in a plant’s life cycle as well, and this holds true for pathogenic, non-beneficial bacteria, as a study on *Salmonella enteridis* in lettuce showed [230].

These observations are consistent with priority effects, an ecological theory that may play a large role in microbiome assembly. Priority effects refer to the order and timing at which species arrive in an ecosystem; the order and timing play a pivotal role in shaping the succession and stable state of an ecosystem [231]. Early arrivers to an ecosystem may shape its successional trajectory via niche preemption or niche modification [231]. In niche preemption, the early arriver uses up and limits the resources that are available for late arrivers, thus inhibiting late arrivers from establishing [231]. The effects of niche preemption can be weakened by environmental factors, such as a nutrient abundance or temperature, which, respectively, can negate the competitive ability of an early arriver or affect the metabolism of competitors in ecological succession [232]. Niche modification, on the other hand, is the alteration by early arrivers of niches that will be available for late arrivers; thus, this effect can be either inhibitory or facilitative for different community members [231]. In microbiome assembly, niche modification may involve the catabolism of large organic molecules into smaller molecules, which may facilitate the establishment of microbial members that rely on the early arriver’s metabolic byproducts [232].

Priority effects likely play an important and practical role in developing inoculation protocols for vertical farming systems. A study on the legume *Medicago lupulina* found that inoculations with an effective *Ensifer* strain followed by an ineffective *Ensifer* strain improved plant growth, compared to the same inoculation with the order reversed [233]. Another study on the legume *Medicago truncatula* found that priority effects—namely plant age, inoculation order, and inoculation synchrony—strongly determined a successful colonization by either a mutualistic rhizobium or a pathogenic nematode [234]. Whether or not priority effects can negate the importance of the aforementioned factors in determining successful PGPB colonization is an open question that should receive further research.

## 5. Conclusions

Using beneficial bacteria to improve plant productivity is an area of research that has received much attention. However, the contexts in which plant–bacterial relationships can thrive remain an area of active research, and beneficial plant bacteria can have variable effects in open-field agriculture. We have described the different ways by which plant–bacterial associations can occur and the many functions provided by beneficial plant bacteria, including nutrient acquisition, phytohormonal control, and abiotic and biotic stress relief.

Vertical farming systems are also gaining much attention as a viable alternative to traditional field agriculture. Although there are certain advantages over soil-based agriculture, vertical farming also has a unique set of challenges and properties that can influence the use of plant-beneficial bacteria.

## 6. Future Directions

With technologies such as vertical farming gaining more attention, it is increasingly important to investigate the various factors in these farming systems that can influence the effectiveness of plant-beneficial bacteria. For example, how might different soilless cultivation techniques—using different substrates and methods of nutrient delivery—change the microbiome of a host plant? How might inoculations with commercial biostimulant microbes affect plant growth in different systems? Furthermore, what are some of the genetic or molecular mechanisms underlying these interactions? As these interactions are elucidated, commercial biostimulant products can earn greater confidence for use in traditional field agriculture and vertical farming systems alike.

## Figures and Tables

**Table 1:** A list of plant–endophyte partnerships for which the rhizophagy process has been documented.

Plant Host	Endophytic Partner	Function	Reference
*Solanum lycopersicum*	*Micrococcus luteus*	Improved seedling growth.	[24]
*Arabidopsis thaliana*	*Escherichia coli*	Increased expression of cell wall modification genes. Downregulation of heat shock proteins.	[25,31]
*Leersia oryzoides/Oryza sativa*	*Pseudomonas* sp. *Pantoea* sp	Improved root gravitropism. Improved root and shoot growth. Improved root hair formation.	[32]
*Phragmites australis/Poa annua*	*Pseudomonas* sp.	Improved seed germination. Improved root branching.	[24]
*Poa reptans*	*Pseudomonas fluorescens*	Production of ethylene. Improved root cell growth.	[26]
*Panicum virgatum*	*Burkholderia* sp.	Nitrogen fixation.	[33]
*Gossypium sp.*	*Bacillus amyloliquefaciens*	Improved seedling growth. Increased expression of nitrate transport genes.	[34,35]
*Vanilla phaeantha*	*Bacillus amyloliquefaciens*	Fungal inhibition. Improved seedling growth.	[36]
*Saccharum officinarum x spontaneum* L.	*Burkholderia australis*	Nitrogen fixation. Improved seedling growth.	[37]
*Hedera helix*	*Bacillus amyloliquefaciens*	IAA synthesis. Fungal inhibition via lipopeptide production.	[38]
*Digitaria ischaemum*	*Pantoea* sp.	Antagonism of competitor Taraxacum officinale.	[39]
*Cynodon dactylon*	*Bacillus* sp.	Improved root hair formation.	[40]
*Saccharum officinarum*	*Gluconacetobacter diazotrophicus*	Nitrogen fixation. Phytohormone production. Siderophore production. Bacteriocin production.	[41]

**Table 2:** A summary of considerations for different staple types of traditional, hydroponic, and vertical farming systems.

Factor	Type of Agriculture				References
**Monetary or technological** **investment**	**Soil-based, field**	**Hydroponic,** **glasshouse**	**Vertical, glasshouse**	**Vertical, CEA**	[155]
	Low	Medium	High	Highest	
**Energy use**	Low	Medium	High	Highest	[156,157,158,159]
**Potential crop productivity**	Lowest	Medium	High	Highest	[156,157]
**Considerations for farm** **placement**	-Climate -Soil fertility -Access to sunlight -High amount of acreage	-Climate -Access to sunlight -High amount of acreage	-Climate -Access to sunlight -Lower amount of acreage	-Lower amount of acreage	[154,155]
**Crop traits that limit** **feasibility**	-None	-Extensive roots -Tall height	-Extensive roots -Tall height -Slow growth -Low ratio of marketable plant parts	-Extensive roots -Tall height -Slow growth -Low ratio of marketable plant parts	[13]
**Commonly** **produced crops**	-Any	-Lettuce -Tomatoes -Herbs -Microgreens -Other leafy greens	-Leafy greens -Microgreens	-Leafy greens -Microgreens	[155,160]

**Table 3:** Advantages and challenges of vertical farming using a closed-loop hydroponics.

Issues	Advantages	Challenges	References
**Water Use**	-No soil runoff in closed hydroponic systems. -Improved water use efficiency.	-Production can be constrained by freshwater resources.	[165,168,188,189]
**Nutrition and** **Fertilization**	-Fewer nutrients wasted to runoff. -Fine control of nutrient concentrations.	-Closed loop systems can increase the risk of nutrient toxicity, if mismanaged.	[57,165,166,167]
**Disease and pests**	-Exclusion of pests, pathogens from closed environments. -Sanitation of tools, equipment, growing area.	-High humidity and temperature may be suitable for pathogens. -Rapid spread if pathogen is not excluded.	[57,164,179]
**Crop productivity**	-Consistent, high yields, depending on crop.	-Major staple crops (rice, wheat, corn) are not feasible to grow in a vertical farm.	[13,165]
**Costs**	-Produce transportation savings and minimization of spoilage. -Reduced pesticide requirements.	-High setup and operational costs.	[159,177]
**Environmental impact**	-Minimization of fertilizer runoff and downstream eutrophication. -Reduced use of synthetic fertilizers and pesticides.	-Wastewater accumulation can be high in salts and organic matter. -Intensive energy use from LEDs.	[175,190]

## Data Availability

Not applicable.
